# Survivin inhibitor YM155 suppresses gastric cancer xenograft growth in mice without affecting normal tissues

**DOI:** 10.18632/oncotarget.6898

**Published:** 2016-01-12

**Authors:** Xiao Jiao Cheng, Jia Cheng Lin, Yan Fei Ding, Liming Zhu, Jing Ye, Shui Ping Tu

**Affiliations:** ^1^ Department of Oncology, Renji Hospital, School of Medicine, Shanghai Jiaotong University, Shanghai, China; ^2^ Department of Gastroenterology, Ruijin Hospital, School of Medicine, Shanghai Jiaotong University, Shanghai, China; ^3^ Pôle Sino-Français de Recherches en Sciences du Vivant et Génomique, Ruijin Hospital, School of Medicine, Shanghai Jiaotong University, Shanghai, China

**Keywords:** YM155, survivin, gastric cancer, xancer stem cells, cancer therapy

## Abstract

Survivin overexpression is associated with poor prognosis of human gastric cancer, and is a target for gastric cancer therapy. YM155 is originally identified as a specific inhibitor of survivin. In this study, we investigated the antitumor effect of YM155 on human gastric cancer. Our results showed that YM155 treatment significantly inhibited cell proliferation, reduced colony formation and induced apoptosis of gastric cancer cells in a dose-dependent manner. Accordingly, YM155 treatment significantly decreased survivin expression without affecting XIAP expression and increased the cleavage of apoptosis-associated proteins caspase 3, 7, 8, 9. YM155 significantly inhibited sphere formation of gastric cancer cells, suppressed expansion and growth of the formed spheres (cancer stem cell-like cells, CSCs) and downregulated the protein levels of β-catenin, c-Myc, Cyclin D1 and CD44 in gastric cancer cells. YM155 infusion at 5 mg/kg/day for 7 days markedly inhibited growth of gastric cancer xenograft in a nude mouse model. Immunohistochemistry staining and Western Blot showed that YM155 treatment inhibited expression of survivin and CD44, induced apoptosis and reduced CD44^+^ CSCs in xenograft tumor tissues *in vivo*. No obvious pathological changes were observed in organs (e.g. heart, liver, lung and kidney) in YM155-treated mice. Our results demonstrated that YM155 inhibits cell proliferation, induces cell apoptosis, reduces cancer stem cell expansion, and inhibits xenograft tumor growth in gastric cancer cells. Our results elucidate a new mechanism by which YM155 inhibits gastric cancer growth by inhibition of CSCs. YM155 may be a promising agent for gastric cancer treatment.

## INTRODUCTION

Gastric cancer is one of the world's most common malignancies. Although diagnosis and treatment of gastric cancers have been greatly improved, the overall survival rate remains low. Less than 20% of patients with gastric cancer survive to 5 years [1]. Survivin, a member of inhibitor apoptosis (IAP) protein family, is implicated in both cell survival and regulation of mitosis in cancer cells [2, 3]. Survivin is expressed in various primary tumors, but is rare expressed in normal differentiated tissues [4]. Previous studies showed that an over expression of survivin is found in human gastric cancer tissues, and is a poor prognostic factor in gastric cancer patients [5-7]. Moreover, overexpression of survivin is detected in gastric cancer cells during drug treatment, indicating that survivin may contribute to chemo-resistance in gastric cancer [8-10]

Recent studies showed that survivin is also associated with cancer stem cells (CSCs). Survivin has been demonstrated to be a downstream gene of the Wnt signal pathway and is highly expressed in colon cancer stem cells [11]. Wnt/*β*-catenin signaling has also been demonstrated to be essential for the proliferation of gastric CSCs [12, 13]. Zhang *et al*. found that mutant APC up-regulates survivin, causing apoptosis inhibition and the expansion of colon tissue stem cells in the colon crypt, thereby initiating colon tumorigenesis [14, 15]. Carter *et al*. showed that survivin is overexpressed in AML stem/progenitor cells [16]. These results suggest that survivin is a potential target for inhibiting cancer stem cells proliferation. Whether YM155 downregulates other target genes of CSC signaling such as β-catenin, c-Myc, Cyclin D1 and CD44 remains unknown. CD44 has been demonstrated to be a marker of human gastric cancer CSC and CD44^+^ gastric cancer cells have stronger tumorigenic than CD44^−^ gastric cancer cells [17, 18]. However, whether YM155 inhibits gastric CSCs remains to be investigated.

Several approaches have been developed to target inhibition of survivin for cancer therapy. Those approaches include, administering a molecular antagonist to target survivin mRNA and to inhibit survivin translation (including antisense oligonucleotides, ribozymes and small interfering RNAs), utilizing gene therapy approaches grounded on the use of survivin dominant-negative mutants (Cys84Ala, Thr34Ala) and the application of the survivin promoter to drive the expression of cytotoxic genes; survivin-based immunotherapy; small-molecule antagonists (suppressing survivin function) [19]. Considerable effects have also been made to target inhibition of survivin for gastric cancer therapy. For example, RNAi-mediated survivin knockdown can effectively inhibit the growth of gastric cancer cells [20, 21]. Our previous studies have shown that suppression of survivin expression or function by plasmid vectors encoding antisense survivin or survivin dominant-negative (DN) mutant (Cys84Ala) can inhibit gastric cancer carcinogenesis and angiogenesis *in vivo* [22]. While those approaches are effective, it is still difficult to use in clinic. Recent studies showed that YM155, a novel small, imidazolium-based compound can specifically inhibit survivin expression and induce apoptosis in human cancer cells [23]. Preclinical studies demonstrated that three to seven-day continuous infusion of YM155 (1-10mg/kg.d) significantly inhibited tumor growth in hormone-refractory prostate cancer, melanoma and non-small-cell lung cancer [24]. Moreover, recent results from completed phase I/II clinical studies show that YM155 was safe at a dose of 4.8 mg/m^2^/day for 168 hours every 3 weeks and exhibited encouraging anti-cancer effect in advanced cancer patients [25-29]. These results suggest that YM155 is a promising agent for cancer therapy.

However, there are no studies to show that YM155 inhibit gastric tumor growth *in vivo*. In this study, we have evaluated the antitumor effect of YM155 in gastric cancer cell lines. We found that YM155 induced apoptosis of gastric cancer cells, inhibited expansion of gastric CSCs and expression of CSC molecules CD44 and β-catanin and suppressed gastric cancer xenograft growth. Our results, for the first time, demonstrate that YM155 inhibits gastric cancer growth by inhibition expansion of CSCs and document a new mechanism that YM155 inhibits tumor growth.

## RESULTS

### YM15 inhibits cell proliferation in gastric cancer cells

**Figure 1:**
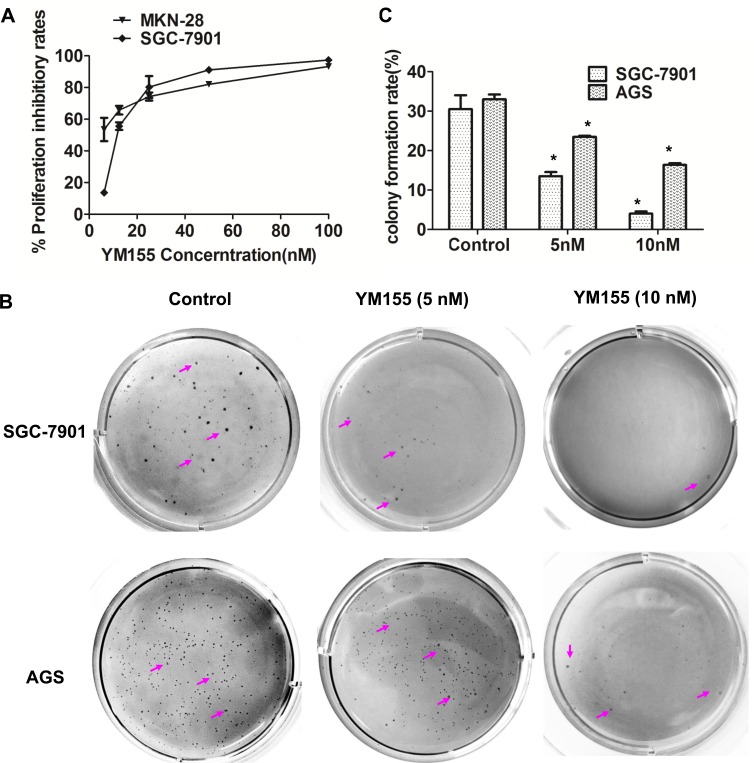
YM155 inhibits anchored-dependent and anchored-independent growth in gastric cancer cells **A.** YM155 inhibits cell proliferation of gastric cancer cells. MKN-28 and SGC-7901 cells were cultured in 96-well plates and treated with YM155 at indicated doses. Cell proliferation was detected by MTT method. The data presented is means ± SD of 3 independent experiments. **B.** YM155 inhibits the colony formation of gastric cancer cells. Colonies in soft agar assay were stained with 0.1% crystal violet at 16 days after culture. Representative colonies were photographed. *Arrows* indicate colonies formed in soft agar. **C.** The number of colonies in **B.** was counted under microscope. Data represented is the means ± SD of three independent experiments. *P < 0.05 compared with the control group.

To investigate the effect of YM155 on cell proliferation of gastric cancer cells *in vitro,* gastric cancer SGC-7901 and MKN-28 cells were treated with YM155 for 48 hours; cell proliferation was measured by MTT. The results showed that YM155 significantly inhibited cell proliferation. The mean IC50 of SGC-7901 and MKN-28 cells were 13.2 nM and 11.6 nM (Figure 1A), respectively. YM155 has also shown a great activity against other gastric cancer cell lines, such as AGS and Hs 764T cell lines, with IC50 values 0.8 nM and 7.3 nM [24].

To further investigated the effect of YM155 on cell transformation. Soft agar assay was conducted to determine cell transformation *in vitro*. The result showed that gastric cancer SGC-7901 cells treated with YM155 formed less number of colonies and smaller size colonies in soft agar compared to control cells treated with DMSO (Figure 1B, upper panel). Same results were obtained in AGS cells (Figure 1B, bottom panel). Quantification analysis showed that YM155 treatment significantly inhibited colony formation in gastric cancer SGC-7901 and AGS cells in a dose-dependent manner (Figure 1C). The rates of colony formation in SGC-7901 were reduced by 55.7% and 86.9% at dose of 5 nM and 10 nM of YM155, respectively. Similar results were obtained in AGS cells. YM155 at 5 nM and 10 nM reduced colony formation of AGS cells by 28.9% and 50.3%, respectively, compared with control DMSO treatment. These results suggest that YM155 inhibits anchored-dependent and anchored-independent growth of gastric cancer cells.

### YM155 induces apoptosis of gastric cancer cells

**Figure 2:**
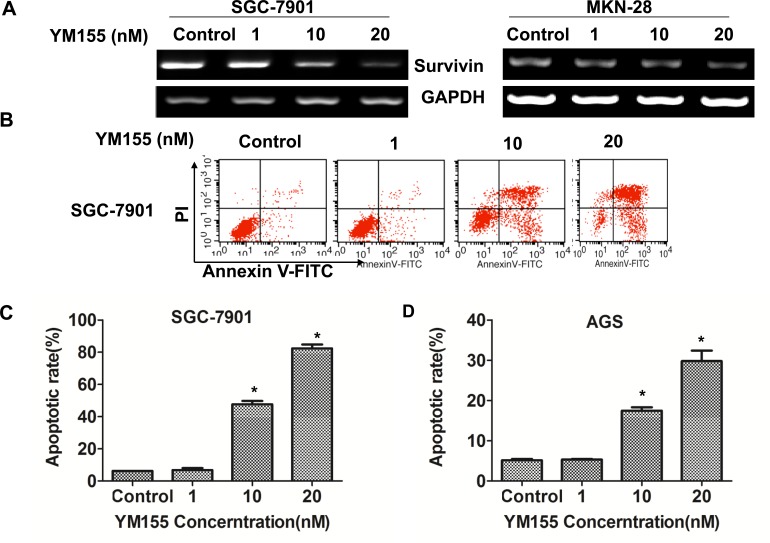
YM155 down-regulates survivin expression and induces apoptosis of gastric cancer cells **A.** YM155 inhibits mRNA expression of survivin. SGC-7901 & MKN-28 cells were treated with YM155 at indicated dose for 24 hours. The mRNA expression of survivin was determined by RT-PCR. **B.**-**D.** YM155 induces apoptosis of gastric cancer cells. SGC-7901 cells and AGS cells were treated with YM155 at indicated doses for 24 hours. Apoptosis was determined by FACS analysis. Representative FACS Plots was shown **in B.** The apoptotic rates are the means ± SD of 3 independent experiments * *P* < 0.05 compared with the control group.

To investigate the effect of YM155 on apoptosis of gastric cancer cells, we first determined the effect of YM155 on survivin expression. The results showed that YM155 effectively inhibited mRNA expression of survivin in SGC-7901 and MKN-28 cells in a dose-dependent manner (Figure 2A). FACS analysis showed that YM155 induced apoptosis of SGC-7901 (Figure 2B) in a dose-dependent manner. Quantification analysis showed that apoptosis rates were significantly increased in YM155-treated gastric cancer cells compared to the control groups (Figure 2C), the apoptosis rates were 47.6 ± 2.9% and 82.4 ± 3.4% when SGC-7901 cells treated by YM155 at the doses of 10 nM and 20 nM YM155 for 24 hours, respectively. The apoptosis rate of control group was only 6.2 ± 0.2%. Same results were obtained in AGS cells (Figure 2D). The results demonstrate that YM155 induce apoptosis of gastric cancer cells efficiently.

### YM155 induces apoptosis by activating intrinsic and extrinsic apoptotic pathways

**Figure 3:**
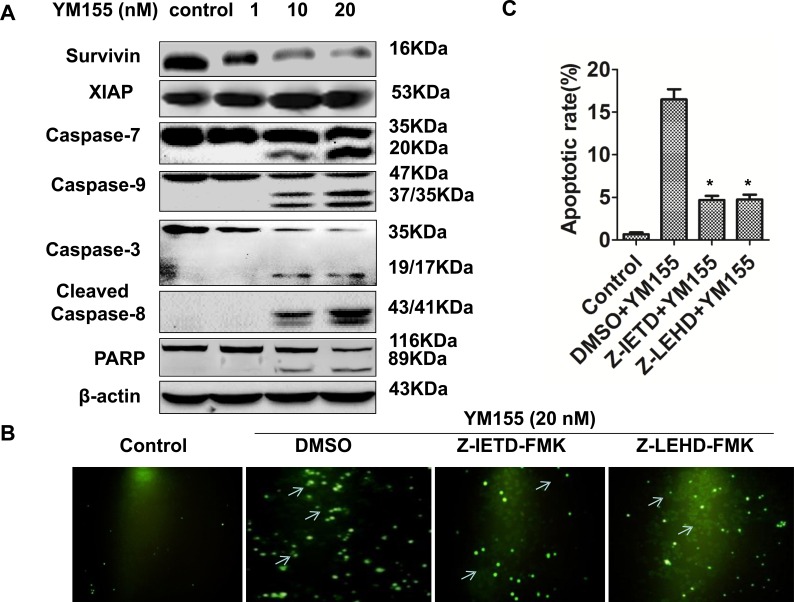
YM155 triggers intrinsic and extrinsic apoptotic pathways **A.** YM155 treatment decreased survivin expression without affecting XIAP and induced cleavage of caspases. Protein expression (caspase-3, -7, -9, -8, PARP) was detected by Western Blot analysis. **B.** Pre-treatment of caspase inhibitors attenuates YM155-induced apoptosis. Gastric cancer cells were pre-treated with 20 μM caspase-8 inhibitor (Z-IETD-FMK) or 20 μM caspase-9 inhibitor (Z-LEHD-FMK) for 1 hour and treated with YM155 (20 nM) for another 24 hours. Apoptosis was detected by TUNEL assay. Representative photos were taken under fluorescence microscope 24 hours after the YM155 treatment. *Arrows* indicate apoptotic cells (green cells) (200 ×). **C.** Quantification of apoptotic cells. The apoptotic rates presented from **B.** are the means ± SD of 3 independent experiments.* *P* < 0.05 compared with the control group.

To investigate the underlying mechanisms by which YM155 induces apoptosis in gastric cancer cells, we determined the effect of YM155 on caspases signaling. Western Blot showed that YM155 could decrease protein level of survivin in a dose-dependent manner without affecting the level of XIAP (Figure 3A). Accordingly, YM155 treatment significantly increased the cleavage of effector caspases such as caspase-3, caspase-7, and PARP (the substrate of caspases 3). Furthermore, YM155 treatment could increase the cleavage of both caspase-9 and caspase-8, which are involved in intrinsic and extrinsic apoptosis signal pathways, respectively (Figure 3A).

To further confirm whether YM155 induces apoptosis through activation of intrinsic and extrinsic apoptosis pathways, we tested the effects of caspase-8 and caspase-9 inhibitors on YM155-induced apoptosis. TUNEL results showed that pre-treatment with caspase-8 inhibitor Z-IETD-FMK (20 M) or caspase-9 inhibitor Z-LEHD-FMK (20 μM) for 1 hour significantly decreased YM155-induced apoptosis of SGC-7901 cells (Figure 3B). Quantification analysis showed that the apoptosis rate of SGC-7901 cells in YM155 treated group was 17.2% ± 7.2%, while pretreated with 20 M caspase-8 inhibitor or caspase-9 inhibitor for 1 hour, the apoptosis rates of SGC-7901 cells were 4.9% ± 1.8% and 5.2 ± 1.4%, respectively (Figure 3C), suggesting that caspase-8 and caspase-9 inhibitors inhibit YM155-induced apoptosis. These results demonstrate that YM155 induced apoptosis by triggering intrinsic and extrinsic apoptotic pathways.

### YM155 inhibits stem-like sphere formation

**Figure 4:**
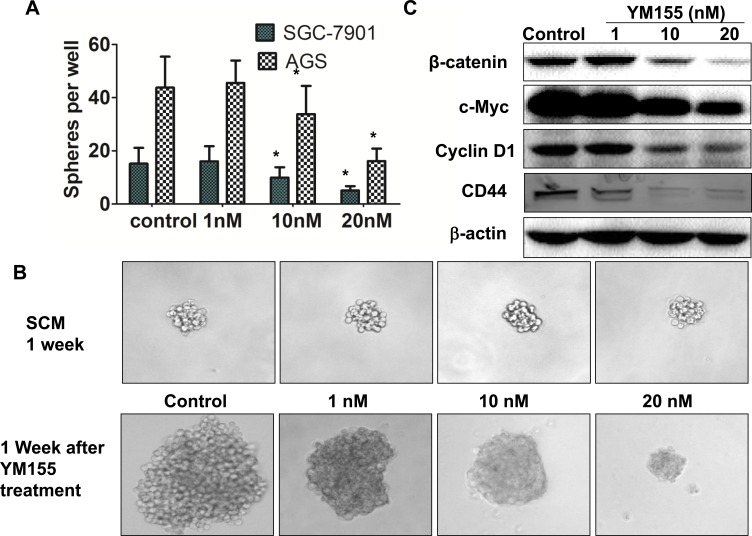
YM155 inhibits formation and expansion of gastric cancer spheres **A.** YM155 inhibits sphere formation of gastric cancer cells. SGC-7901 and AGS cells (100 per well) were cultured for 2 weeks in SCM supplement with or without YM155 at indicated doses. The spheres were counted under microscope. The data presented are the means ± SD of 3 independent experiments.* *P* < 0.05 compared with the control group. **B.** YM155 inhibits expansion of spheres of gastric cancer cells. SGC-7901 cells were cultured for 1 week in SCM to form sphere (upper panel), and then treated with YM155 at indicated doses for another 1 week. YM155 treatment inhibits growth of spheres. Representative photos were taken at one week after the YM155 treatment (200 ×). **C.** YM155 inhibited expression of CSC molecules. SGC-7901 cells were treated with YM155 at indicated doses for 24 hours. The protein levels of genes were determined by Western Blot.

Overexpression of survivin has been shown to be associated with CSCs. Hypothesized that YM155 can suppress gastric CSCs by inhibition of survivin. Several approaches have been developed to evaluate CSCs [30-32]. One approach is to use a *in vitro* method, “spheroid colony formation”, that candidate CSCs were cultured in serum-free medium containing only EGF and bFGF (stem cell medium, SCM) using a ultra-low-attachment plates. We first investigated the effect of YM155 on sphere formation. SGC-7901 cells and AGS cells were cultured in SCM with and without YM155 for 2 weeks. Quantification analysis of spheres showed that YM155 reatment significantly reduced spheres formation in SGC-7901 and AGS cells in dose dependent patterns (Figure 4A). These results indicate that YM155 can inhibit formation of spheres in gastric cancer cells. To determine whether YM155 also reduces expansion of gastric CSCs, SGC-7901 cells were first cultured in SCM for one week to form sphere. One week after culturing, SGC-7901 cells formed small spheroid colonies (spheres) (Figure 4B, upper panel). These formed spheres of SGC-7901 cells were then treated with YM155 at the doses of 1 nM, 10 nM and 20 nM in SCM for one more week. We observed that vehicle-treated spheres greatly expanded and formed large size colonies, and that YM155-treated spheres grew slowly, and formed smaller size colonies compared to vehicle-treated spheres (Figure 4B, bottom panel). The similar inhibition of sphere growth was also observed in AGS cells treated with YM155 (data not shown). The results suggest that YM155 inhibits expansion of gastric CSCs.

### YM155 inhibits expression of CSC molecules

We then explored the underlying mechanisms by which YM155 inhibits the expansion of gastric CSCs. Since Wnt/*β*-catenin signaling has been demonstrated to be essential for the expansion of gastric CSCs [12, 13], we tested the effect of YM155 on Wnt signaling. Western Blot showed that YM155 decreased the level of β-catenin and the expression of c-Myc and Cyclin D1, which are downstream of Wnt/β-catenin signaling (Figure 4C). CD44 has been identified as a marker of gastric CSCs [17, 18, 33]. YM155 treatment downregulated expression of CD44 in gastric cancer cells (Figure 4C). These results suggest that YM155 inhibits expansion of gastric CSCs by inhibiting Wnt/*β*-catenin signaling and expression of CD44.

### YM155 inhibits growth of gastric cancer xenograft

**Figure 5:**
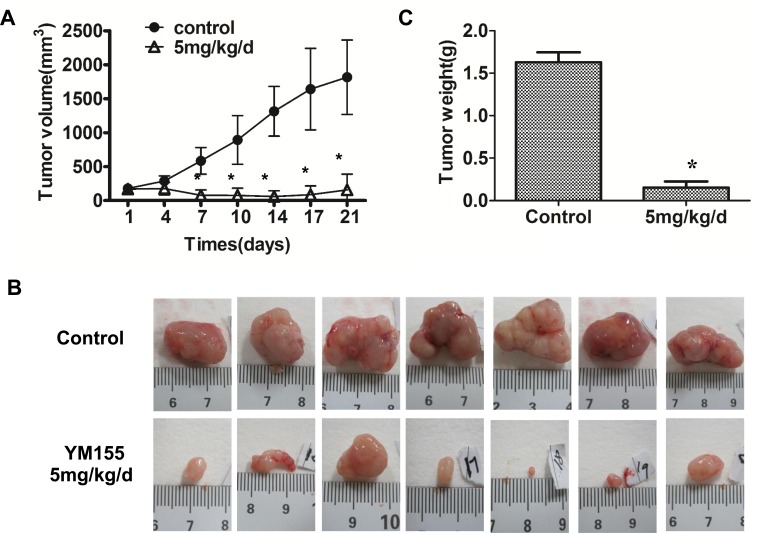
YM155 inhibits xenograft growth of gastric cancer Tumor xenografts were established by s.c. injection of SGC-7901 cells into the right flanks of the mice (5×10^6^/0.1ml per mouse). When tumor size reached around 80 mm^2^−100 mm^2^, mice were administered with a 7-day YM155 continuous infusion using a micro-osmotic pump. Tumor size was measured every three days. The tumor growth curve was shown in **A.** Three weeks after treatment, xenograft tumors were excised and photographed. Representative tumor photos were shown in **B.** Data presented are the means ± SD of tumor weight per mouse (*m* = 7) **C.** *P < 0.05 compared with the control group.

We finally investigated the effects of YM155 on tumor growth in a xenograft mouse model. SGC-7901 cells were injected into the frank of athymic nude mice to establish tumor xenograft. When tumor size reached around 80 mm^3^-100 mm^3^, mice received a 7-day continuous infusion of YM155 at 5 mg/kg/day or vehicle control. The results showed that tumors in the mice treated with YM155 grew slowly compared to tumor in the mice treated with vehicle control (Figure 5A-5B). Tumor growth curve showed that YM155 treatment resulted in persistent inhibition of tumor growth and tumor shrank (Figure 5B). Accordingly, YM155 treatment significantly reduced tumor weight compared to the control treatment (Figure 5C). The results demonstrate that YM155 inhibits gastric cancer tumor growth *in vivo*.

### YM155 induces apoptosis and inhibits gastric CSCs *in vivo*

**Figure 6:**
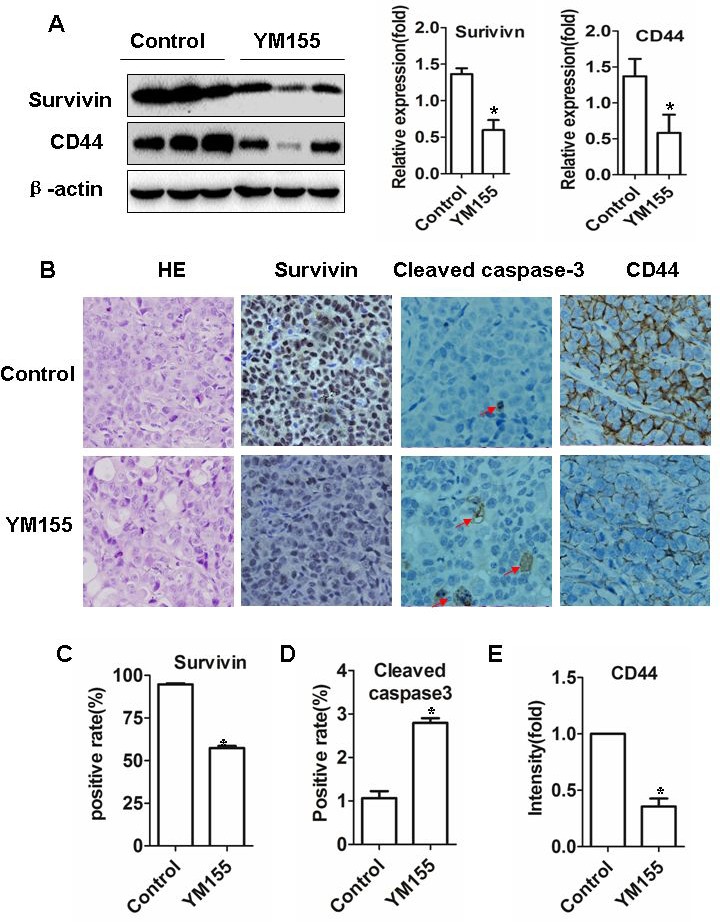
YM155 induces apoptosis and inhibits gastric CSCs *in vivo* **A.** YM155 treatment decreased protein levels of survivin and CD44. Xenograft tumor tissues (from Figure 5) were extracted and protein concentrations were measured. The levels of protein were determined by Western blot (left panel). The data of the densities presented are means ± SD of 3 mice. **B.** YM155 treatment inhibits expression of surviving, cleaved caspase-3 and CD44. Tissue sections were subjected to surviving, cleaved caspase-3 and CD44 immunostaining. Representative photos were shown (Original magnification × 400). **C.**-**D.** Quantification analysis of survivin and cleaved caspase 3 positive staining cells. Data presented are means ± SD of 7 mice. *p < 0.01, compared to control treatment. **E.** Quantification analysis of intensities of CD44 positive staining cells. Data presented are means ± SD of five mice. *p < 0.01, compared to control treatment.

To determine whether YM155 inhibits survivin expression in xenograft tissues, tumor tissues were subjected to Western Blot and IHC staining. Western Blot showed that the band densities of survivin protein were lower in the tumor tissues from YM155-treated mice than in those from vehicle-treated mice (Figure 6A, left and middle panel). IHC showed that the intensity of survivin^+^ staining in tumor tissues from YM155-treated mice were significantly weaker than in those from vehicle-treated mice (Figure 6B). Quantification analysis showed that the number of survivin^+^ staining cells in the tumors from YM155-treated mice were also significantly lower than in those from vehicle-treated mice (Figure 6C). The results suggest that YM155 inhibit survivin expression *in vivo*.

Accordingly, we found that YM155 treatment increased the number of cleaved caspases-3-positive cells (apoptotic cells) in xenograft tumor tissues compared to control treatment (Figure 6B). Quantification analysis showed that caspase 3 positive rates in the tumor was significantly higher in YM155-treated mice than vehicle-treated mice (2.80% ± 0.11% *vs* 1.07 ± 0.15 %, *p* < 0.01) (Figure 6D). These results indicate that YM155 induces apoptosis of gastric cancer *in vivo*.

To further explore whether YM155 also inhibits gastric CSCs *in vivo* we determined expression of CD44 in xenograft tissues by Western Blot and IHC staining. Western blot showed that the protein levels (densities of band) of CD44 in the tumors from YM155-treated mice were lower than those in the mice from vehicle-treated mice (Figure 6A left and right panel). Although IHC staining showed that the number of CD44^+^ staining cells was not significantly different between the groups, the intensity of CD44^+^ staining were markedly weaker in tumor tissues from YM155-treated mice compared to those in tumor tissues from vehicle-treated mice (Figure 6B). Quantification analysis showed that the densities of CD44^+^ positive staining were significantly lower in the tumor from the YM155-treated mice than those in tumor tissues from vehicle-treated mice (Figure 6E). The results indicate that YM155 reduces CD44 expression and inhibits gastric CSCs *in vivo*.

### Assessment of YM155 treatment safety

**Figure 7:**
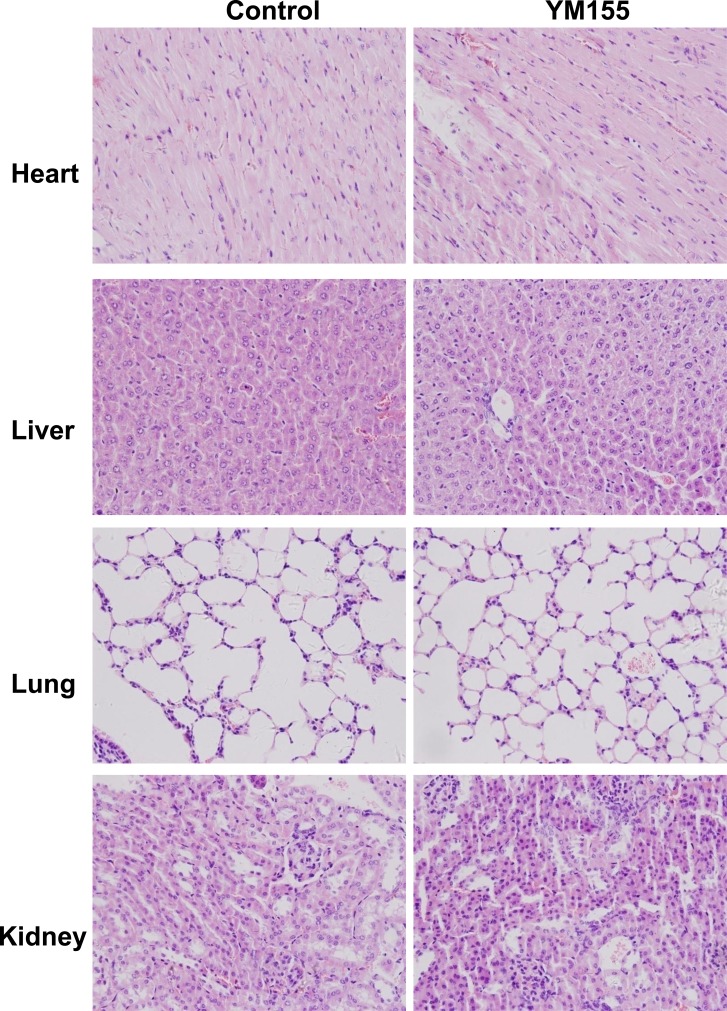
YM155 treatment does not cause histopathological alteration of organs Main organs (heart, liver, lung and kidney) of mice treated with YM155 or control vehicle were subjected to H&E staining. Histology analysis (heart, liver, lung and kidney) were conducted by pathologist. Representative photos were shown (Original magnification × 400).

We also determined the effects of YM155 on normal tissues. Furthermore, we did not observe obvious pathological change in some organs (heart, liver, lung, and kidney) of YM155-treated mice (Figure 7), suggesting that YM155 treatment is safety in our mouse mice model.

## DISCUSSION

Survivin is highly expressed in the majority of human cancer tissues, including gastric cancer, and is associated with drug-resistance and poor prognosis [34, 35]. Suppression of survivin expression or function resulted in spontaneous apoptosis, increased sensitivity to cytotoxic drugs and suppression of tumor growth in a nude mouse model [22, 36]. These data indicate that targeted inhibition of survivin is a potential and promising approach for the treatment of gastric cancer [22]. In this study, we further found that YM155, a specific survivin suppressant [24], induced apoptosis of gastric cancer cells, inhibited expansion of gastric CSCs and reduced expression of CSC molecules (CD44 and β-catanin) and suppressed gastric cancer xenograft growth. Our results, for the first time, demonstrate that YM155 inhibit gastric cancer growth by inhibition CSCs and document a new mechanism that YM155 inhibits tumor growth.

Our previous studies have demonstrated that inhibition of survivin function by AAV-mediated survivin mutant or inhibition of survivin expression by antisense oligonucleotide suppress gastric and colon cancer growth [37, 38]. Although AAV vector can efficaciously mediated long-term expression of survivin mutant, its safety and inconvenience are still the obstacles for its clinical use. YM155 is a small imidazolium-based molecule that inhibits specifically both mRNA and protein expression of survivin [24]. YM155 has been demonstrated to suppress the growth of a large number of human cancers including non-small-cell lung cancer, breast cancer, hormone-refractory prostate cancer, ovarian cancer, leukemia, non-Hodgkin's lymphoma, sarcoma, melanoma, esophageal cancer, renal cancer, breast cancer, hepatoma [24, 39-42]. The combination of YM155 and chemotherapeutic drugs (including etoposide, platinum, doxetaxel) enhanced inhibition of survivin expression, resulting in stronger inhibition of tumor growth in some cancer types such as non-small cell lung cancer, melanoma and neuroblastoma. The mean log growth inhibition of 50% (GI50) value was around 15 nM for YM155 [43-45]. In this study, we have demonstrated the potent anti-proliferation activity of YM155 to gastric cancer cells with the mean IC50 of YM155 was 11.6∼13.2 nM. The data suggest that gastric cancer cells are also sensitive to YM155. Furthermore, we showed that YM155 significantly inhibited gastric xenograft tumor growth in a nude mouse model and reduced tumor weight, exhibiting strong anti-tumor effect. Our results demonstrated that survivin inhibitor, YM155, is able to inhibit gastric cancer growth.

Survivin has been demonstrated to inhibit caspase 9 and caspase 3, resulting in inhibition intrinsic apoptosis pathway [46]. Previous studies have suggested that YM155 specifically down regulates survivin expression in prostate cancer cells and non-small cell lung cancer (NSCLC) cells without affecting other proteins [23, 47]. Our results showed that YM155 downregulated survivin expression without affecting XIAP and induced cleavage of caspase 3, caspase 9 and caspase 8. Furthermore, the inhibitors of caspases 9 and caspase 8 significantly suppressed YM155-induced apoptosis, suggesting that YM155 induces apoptosis by activating intrinsic and extrinsic apoptotic pathway. A study showed that YM155 not only inhibits survivin, but also regulate expression of a large number of genes including in death receptor signaling and TNFR1 signaling that induce apoptosis through a extrinsic apoptosis pathway [48], consistent with our results.

While YM155 is originally identified as a survivin inhibitor, its mechanisms of antitumor actions are not fully understood. Recently, a number of studies have shown that YM155 could inhibit expression of EGFR, XIAP, Mcl-1 [49-51], suggesting that YM155 function is beyond to inhibition of survivin. We found that YM155 could inhibit expansion of gastric CSCs. CSCs are a subpopulation in tumors that have features to initiate tumor growth, sustain tumor self-renewal, promote relapse and metastasis by giving rise to new tumors [52]. However, the roles of CSCs in tumorigenesis remain in debate [53, 54]. Survivin is highly expressed in colon CSCs [11] and is upregulated by interleukin-4 [55]. Mutant APC up-regulates survivin, causing apoptosis inhibition and the expansion of colon tissue stem cells in the colon crypt, thereby initiating tumorigenesis [14, 15]. Survivin has been shown to be overexpressed in AML stem/progenitor cells [16]. The data suggested that survivin plays a role in regulation of CSCs. It is well known that Wnt/β-catenin signaling is essential for expansion of CSCs [12, 13]. Survivin has been shown to be a downstream gene of Wnt signaling pathway [11]. Therefore, survivin is a potential targeting for inhibition of CSCs. In this study, we observed that YM155 markedly suppressed formation and expansion of spheres in gastric cancer cells and downregulated expression of β-catenin, c-Myc and Cyclin D1, which are downstream genes of Wnt/β-catenin signaling. More importantly, YM155 inhibited CD44 expression in gastric cancer cells and gastric cancer tissues both *in vitro* and *in vivo*. CD44^+^ gastric cancer cells is a CSC marker of human gastric cancer and have stronger tumorigenic than CD44^−^ gastric cancer cells [17, 18]. Our results, for the first time, show that YM155 inhibits gastric tumor growth by inhibition of expansion of CD44^+^ gastric CSCs.

YM155 has been studied in clinical trial in several cancer types. In phase I and II studies, YM155 has shown to be safe and well-tolerated, with a maximum tolerated dose of 8.0 mg/m^2^/d. In our xenograft mouse model, a 7-day continuous infusion of YM155 at 5 mg/kg induced massive tumor regression with decreased expression of survivin and CD44 in intratumoral. Furthermore, we did not observe serious side effects in some organisms. The data document that YM155 is a safety and promising agent for gastric cancer therapy.

## MATERIALS AND METHODS

### Cell lines and regents

The human gastric cancer cell lines SGC-7901, MKN28 and AGS were maintained in RPMI1640 (Thermo Scientific, China) supplemented with 10% fetal bovine serum (Life Technologies inc., Grand island, USA), 100 U/ml of penicillin sodium, and 100 mg/ml of streptomycin sulfate at 37°C in a humidified air atmosphere containing 5% CO2. YM155 monobromide (YM155) was purchased from ChemieTek Company (Indianapolis, USA).

### MTT assay

*In vitro* cell proliferation was measured using MTT assay. Cells in the logarithmic phase of growth were seeded into 96-well culture plates at 1× 10^4^ cells per well for 24 hours. After treatment with different concentrations of YM155 or vehicle control for 48 hours, 100 μl of MTT solution (1 mg/ml) were added to each well, and the cells were further incubated at 37°C for 4 hours. The supernatant was replaced with dimethyl sulfoxide (DMSO) to dissolve formazan production. The absorbance at wave length 595 nm was measured using micro-ELISA reader (Bio-Rad, Hercules, CA). The mean 50% growth inhibition (IC50) value was calculated by logistic analysis. The mean IC50 value was obtained from the results of 3 independent assays.

### Soft agar colony formation assay

Anchorage-independent cell growth was determined by soft agar clone formation assay. Cells (5 × 10^2^) in 0.5 ml complete culture medium containing 0.3% agar and YM155 or vehicle were cultured on the top of 0.7% agar in the same medium. Dishes were then transferred into culture incubator. Cultures were stained with 0.1% crystal violet at 16 days, colonies were counted under microscope (magnification 50 ×). Colonies containing ≥ 50 cells were considered viable.

### Flow cytometry assay for apoptosis

Cells were collected and washed with cold PBS, then stained with FITC Annexin V/PI (BD Biosciences, San Diego, USA) according to production manuals. Apoptotic cells were analyzed by flow cytometry (Coulter, Luton, United Kingdom).

### Western blot analysis

Cells were lysed in lysis buffer (1X PBS,1% NP40,0.1% SDS,5mM EDTA,0.5% sodium deoxyccholate, 1 mM sodium orthovanadate, and 1 mM phenylmethylsulfonyl fluoride). Protein samples were electrophoresed in 10% denaturing sodium dodecylsulfate polyacrylamide gel, and transferred to Immobilon-P transfer membrane (Millipore, Billerica, MA). The blots were incubated with specific primary antibodies, reacted with a peroxidase-conjugated second antibody (Cell Signaling Technology, Inc., Danvers, USA) and then visualized by chemiluminescence HRP Substrate (Millipore, Billerica, MA). Survivin (71G4B7), PARP, Caspase 3/9/7, cleaved caspase-8, β-catenin, c-Myc, Cyclin D1, CD44 antibodies were all purchased from Cell Signaling, (Cell Signaling Technology, Inc., Danvers, USA) and β-actin antibody was from Santa Cruz Biotechnology (Santa Cruz, California).

### Polymerase chain reaction (PCR)

Total RNA from cultured cells was prepared with RNAprep pure cell kit (Tiangen). First-strand cDNA was synthesized with oligo (dT) primer by using PrimeScript^®^ RT reagent Kit (Takara, Daliang, China). Primer sequences were designed using the Primer Quest^SM^ and were as follows: human survivin (forward) 5-ATGGGTGCCCCGACGTTGCC-3 and (reverse) 5-TCAATCCATGGCAGCCAG CT-3; human CD44 (forward) 5-AAAGCAGGACCTTCATCCCAGTGA-3 and (reverse) 5-TTGCTCCACCTTCTTGACTCCCAT-3; human GAPDH (forward) GAAGACTGTGGATGGCCCCT and (reverse) GTCCACCACCCTGTTGCTGT. PCR was performed using the PCR Master Mix (Tiangen, China), according to the manufacturer's instructions.

### *In situ* detection of apoptotic cells by TUNEL assay

Cells were harvested and fixed by 0.4% paraformaldehyde, TUNEL staining was carried out using In Situ Cell Death Detection Kit (Roche, Indianapolis, USA) according to the manufactory manuals. Apoptotic cells show strong nuclear green fluorescence. The apoptotic cells were counted in 5 randomly selected fields viewed at × 20 magnification. The apoptotic index was calculated as the number of apoptotic cells/total number of nucleated cells ×100%.

### Spheroid colony formation assay

Gastric cancer cells (10^2^ cells per well) were inoculated in a ultra-low-attachment 96-well plates (Corning, USA) supplemented with 200 μl of RPMI-1640 medium (Thermo Scientific, China) plus 0.3% albumin from bovine serum (BSA, Sigma-Alorich, USA), 20 ng/ml human recombinant epidermal growth factor (EGF, Invitrogen Corp., Frederick, MD, USA) and 10 ng/ml human recombinant basic fibroblast growth factor (bFGF, Invitrogen Corp., Frederick, MD, USA). Two weeks after culture, each well was examined under light microscope and the total spheroid colonies numbers per well were counted. The rates of sphere formation were calculated as the number of apoptotic cells/total number of nucleated cells 1×100%.

### Gastric cancer xenograft experiment

Five weeks old female BALB/c nude mice were purchased from Shanghai Experimental Animals Centre of Chinese Academy of Sciences. All animal studies abode the rules of The Laboratory Animal Ethics Committee of Renji Hospital, Shanghai Jiaotong University School of Medicine. Xenografts were established by *s.c.* injection of SGC 7901 cells into the right flanks of the mice (5×10^6^ /0.1ml per mouse. After one week, tumor size reached approximately 80 mm^3^-100 mm^3^, mice were randomly assigned to treatment and control groups. YM155 at 5mg/kg/day or vehicle control was administered subcutaneously as a 7-day continuous infusion using a micro-osmotic pump (Alzet model 1007D) implanted in the left dorsal flank under anesthesia. Body weight of mice was assessed twice weekly, and tumor diameter was measured using standard calipers. After two weeks observation, mice were sacrificed and tumors were weighed, subsequently processed for immunohistochemistry and Western Blot.

### Immunohistochemistry

The expression of survivin, CD44, cleaved-caspase-3 in tumor tissues was detected with the Ultra-Sensitive^TM^S-P (Mouse/Rabbit) kit (Maxin-Bio, China) according to the manufacturer instructions. Briefly, sections were dewaxed in xylene. Antigen retrieval was performed with a microwave for 10 min at 100°C. The sections were then incubated with rabbit anti-survivin antibody (1:200), anti-CD44 antibody (1:50), anti-cleaved-caspase-3 antibody (1:100) (cell signaling) for 1 hour, followed by biotinylated anti-IgG Antibody and streptavidin-biotinylated-complex horseradish peroxidase. Antibody binding was visualized by incubated with DAB (Maxin-Bio, China). Sections were then stained with hematoxylin.

### Statistical analysis

Data were expressed as the means of at least three different experiments ± SD. The results were analyzed by ONE-WAY ANOVA, and *P* ≤ 0.05 was considered statistically significant.
